# A Large Population Histology Study Showing the Lack of Association between ALT Elevation and Significant Fibrosis in Chronic Hepatitis B

**DOI:** 10.1371/journal.pone.0032622

**Published:** 2012-02-28

**Authors:** Wai-Kay Seto, Ching-Lung Lai, Philip P. C. Ip, James Fung, Danny Ka-Ho Wong, John Chi-Hang Yuen, Ivan Fan-Ngai Hung, Man-Fung Yuen

**Affiliations:** 1 Department of Medicine, The University of Hong Kong, Queen Mary Hospital, Hong Kong; 2 Department of Pathology, The University of Hong Kong, Queen Mary Hospital, Hong Kong; 3 State Key Laboratory for Liver Research, The University of Hong Kong, Queen Mary Hospital, Hong Kong; The Chinese University of Hong Kong, Hong Kong

## Abstract

**Objective:**

We determined the association between various clinical parameters and significant liver injury in both hepatitis B e antigen (HBeAg)-positive and HBeAg-negative patients.

**Methods:**

From 1994 to 2008, liver biopsy was performed on 319 treatment-naïve CHB patients. Histologic assessment was based on the Knodell histologic activity index for necroinflammation and the Ishak fibrosis staging for fibrosis.

**Results:**

211 HBeAg-positive and 108 HBeAg-negative patients were recruited, with a median age of 31 and 46 years respectively. 9 out of 40 (22.5%) HBeAg-positive patients with normal ALT had significant histologic abnormalities (necroinflammation grading ≥7 or fibrosis score ≥3). There was a significant difference in fibrosis scores among HBeAg-positive patients with an ALT level within the Prati criteria (30 U/L for men, 19 U/L for women) and patients with a normal ALT but exceeding the Prati criteria (p = 0.024). Age, aspartate aminotransferase and platelet count were independent predictors of significant fibrosis in HBeAg-positive patients with an elevated ALT by multivariate analysis (p = 0.007, 0.047 and 0.045 respectively). HBV DNA and platelet count were predictors of significant fibrosis in HBeAg-negative disease (p = 0.020 and 0.015 respectively). An elevated ALT was not predictive of significant fibrosis for HBeAg-positive (p = 0.345) and -negative (p = 0.544) disease. There was no significant difference in fibrosis staging among ALT 1–2×upper limit of normal (ULN) and >×2 ULN for both HBeAg-positive (p = 0.098) and -negative (p = 0.838) disease.

**Conclusion:**

An elevated ALT does not accurately predict significant liver injury. Decisions on commencing antiviral therapy should not be heavily based on a particular ALT threshold.

## Introduction

Clinical course of chronic hepatitis B (CHB) is highly variable, ranging from an asymptomatic carrier state [1], [2] to the development of cirrhosis, hepatic decompensation and hepatocellular carcinoma (HCC) [3]. The aim of CHB treatment is to prevent the development of these long-term complications, of which effective implementation would require identification of high-risk patients. Histologic injury and fibrosis have a good correlation with long-term risk, since repeated hepatitic flares in CHB were found to have increased necroinflammation on liver biopsies, resulting in increased fibrogenesis and eventual disease progression [4].

Serum alanine aminotransferase (ALT) is an important biochemical marker used in the assessment of hepatic injury, but is limited by its poor correlation with disease severity [5]. Serum HBV DNA levels directly reflect the degree of HBV replication, and are strongly correlated with long-term mortality [6], [7]. However, patients in the immune tolerant phase have minimal changes on liver biopsy despite high HBV DNA levels [8]. Although liver biopsy remains an integral part in determining disease severity , it is an invasive procedure and not without complications [9]. Sampling error and intra-observer variations are also unavoidable [10], [11].

Current treatment guidelines recommend the use of both serum ALT and HBV DNA in selecting patients for therapy, with a persistent ALT level of more than 2×upper limit of normal (ULN) required for commencing therapy in two of the three international treatment guidelines on CHB [12], [13]. Certain subgroups still require consideration of liver biopsy. These include hepatitis B e antigen (HBeAg)-positive patients of 40 years and older with ALT 1–2×ULN, and HBeAg-negative patients with ALT 1–2×ULN and an elevated HBV DNA (more than 1×10^4^ copies/mL or 2000 IU/mL). A study of 4376 HBeAg-negative patients from Taiwan claimed that normal ALT levels in HBeAg-negative disease had good prognosis [14]. However, other studies have shown that between 10 to 37% of CHB patients with normal ALT already having significant necroinflammation, fibrosis and even cirrhosis on liver biopsy [10], [15], [16]. An Italian population study by Prati et al based on 6835 healthy individuals suggested to lower the ULN of ALT to 30 U/L for men and 19 U/L for women [17], making the definition of “normal ALT” more confusing.

Previous studies investigating the association between clinical parameters and advanced histologic abnormalities were limited by either a small sample size or a disproportional distribution of HBeAg-positive and -negative disease [18], [19], [20], [21]. The present study aimed at studying the association between ALT and advanced histologic abnormalities in the 3 phases of HBV replication: the immune tolerant phase, the immune clearance phase and HBeAg-negative disease.

## Methods

From 1994 to 2008, 1054 treatment-naive CHB patients followed up in the Department of Medicine, the University of Hong Kong, Queen Mary Hospital were screened for entry into therapeutic drug trials that required liver biopsy. Three hundred and nineteen out of these 1054 patients with similar demographics and clinical characteristics who had consented to enter these trials were recruited in the present study. In addition, we had previously investigated and reported the usage of routinely available clinical parameters to derive a score to predict significant fibrosis in CHB based on 237 out of these 319 patientss [22].

Patients were recruited if they were positive for hepatitis B surface antigen (HBsAg) for at least 6 months and had available baseline biochemical and hematological parameters on presentation. The inclusion criteria for ALT and HBV DNA for each therapeutic drug trial differed, and are listed in Table 1. Patients who had decompensated liver disease and other concomitant liver disease, including chronic hepatitis C or D virus infection, primary biliary cirrhosis, autoimmune hepatitis, Wilson's disease, and significant intake of alcohol (20 grams per day for female; 30 grams per day for male) were excluded. All patients had written consent on entry into trials for research purposes, and all trials were approved by the Institutional Review Board of the University of Hong Kong.

**Table 1:** Inclusion criteria concerning ALT and HBV DNA for all 319 patients.

HBeAg-positive	88 patients	ALT<10×ULN
		HBV DNA≥1.4×10^6^ copies/mL
	53 patients	ALT 1.3–10×ULN
		HBV DNA≥1×10^5^ copies/mL
	49 patients	ALT 1.3 – 10×ULN
		HBV DNA≥1×10^6^ copies/mL
	15 patients	ALT 1.3 – 10×ULN
		HBV DNA≥3×10^6^ copies/mL
	6 patients	ALT < 10×ULN
		HBV DNA≥1×10^6^ copies/mL
HBeAg-negative	63 patients	ALT 1.3–10×ULN
		HBV DNA≥1×10^4^ copies/mL
	24 patients	ALT 1.3–10×ULN
		HBV DNA≥3×10^6^ copies/mL
	21 patients	ALT 1.3–10×ULN
		HBV DNA≥1×10^6^ copies/mL

ULN = upper limit of normal.

Clinical and biochemical parameters were recorded from all patients at the day of liver biopsy. These include the patient's age, gender, HBeAg status, albumin, bilirubin, ALT, AST, platelet count and HBV DNA level. The ULN of ALT was based on the respective therapeutic drug trials, and was ranged from 45 to 53 U/L in men and 31 to 43 U/L in women. Serum HBV DNA levels were measured by three different assays, as follow: a branched DNA assay (Versant HBV DNA 3.0 assay, Bayer Health-Care Diagnostic Division, Tarrytown, NY), with a lower limit of quantification of 2000 copies/mL (400 IU/mL) in 88 patients, Cobas Amplicor HBV Monitor Test (Roche Diagnostic, Branchburg, NJ) with a lower limit of quantification of 300 copies/mL (60 IU/mL) in 115 patients, Cobas Taqman assay (Roche Diagnostic, Branchburg, NJ) with a lower limit of quantification of 60 copies/mL (12 IU/mL) in 116 patients.

Two different biopsy needles were used to obtain liver biopsies. An 18G sheathed cutting needle (Temno Evolution, Cardinal Health, McGaw Park, IL) was used for 88 patients, of which the biopsies obtained were 1.5 to 1.8 cm in length. For the rest of the cohort, liver biopsies were obtained using a 17G core aspiration needle (Hepafix, B. Braun Melsungen AG, Germany), with a biopsy length between 2 to 5 cm. Only pre-treatment biopsies were included. Biopsies were fixed, paraffin-embedded, and stained with hematoxylin and eosin for morphological evaluation and Masson's trichrome stain for assessment of fibrosis. The pathologist assessing the biopsy specimens was blinded to the biochemical and virologic results of the patients. Histologic grading of necroinflammation and the staging of liver fibrosis were performed using the Knodell histologic activity index (HAI) [23] and Ishak fibrosis score [24] respectively. Necroinflammation was graded from 0 to 18 while fibrosis was staged from 0 to 6. Significant necroinflammation was defined as a Knodell HAI of 7 or more. Significant fibrosis was defined as an Ishak score of 3 or more, meaning the presence of bridging fibrosis or cirrhosis.

All statistical analyses were performed using SPSS version 16.0 (SPSS Inc, Chicago, Illinois). The Mann-Whitney U-test was used for continuous variables with a skewed distribution; Pearson's chi squared test was used for categorical variables. Multivariate logistic regression was used to determine whether the identified variables associated with advanced histologic abnormalities were independent risk factors. A two-sided p value of <0.05 was considered statistically significant.

## Results

The baseline characteristics of the study population are depicted in Table 2. Two hundred and eleven patients were HBeAg-positive, of which 40 , 78 and 93 patients had a normal ALT, ALT 1–2×ULN and ALT more than 2×ULN respectively. Of the 108 HBeAg-negative patients, only four (3.7%) had normal ALT levels.

**Table 2:** Baseline Characteristics of all 319 patients.

Patient Characteristic	HBeAg-positive	HBeAg-positive	HBeAg-negative
	Normal ALT	Elevated ALT	(n = 108)
	(n = 40)	(n = 171)	
Age	32 (17–43)	32 (16–60)	46 (18–63)
Male	62.5% (n = 25)	67.3% (n = 115)	71.3% (n = 77)
Albumin (g/L)	47 (43–52)	45 (29–55)	46 (40–53)
Bilirubin (umol/L)	9 (5–22)	11 (3–31)	13 (3–28)
ALT (U/L)	27 (14–55)	93 (38–636)	90 (41–576)
≤1×ULN	100% (n = 40)	-	3.7% (n = 4)
1–2×ULN	-	45.6% (n = 78)	48.1% (n = 52)
>2×ULN	-	54.4% (n = 93)	48.1% (n = 52)
AST (U/L)	23 (15–39)	56 (23–304)	55 (26–255)
Platelet (×10^9^/L)	211 (148–308)	166 (89–334)	189 (80–331)
HBV DNA (log IU/mL)	8.14 (4.83–10.96)	7.89 (3.48–14.00)	5.71 (2.67–9.42)

Continous variables expressed in median (range).

ULN = Upper limit of normal.

**Figure 1:**
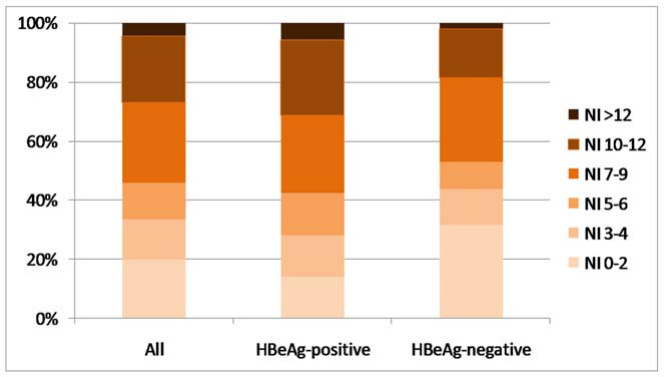
Distribution of liver necroinflammation (graded by Knodell histologic activity index) among 319 chronic hepatitis B patients.

**Figure 2:**
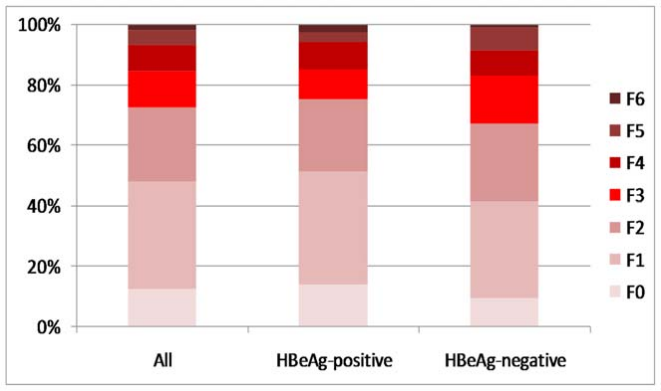
Distribution of liver fibrosis (staged by Ishak fibrosis score) in 319 chronic hepatitis B patients.

**Figure 3:**
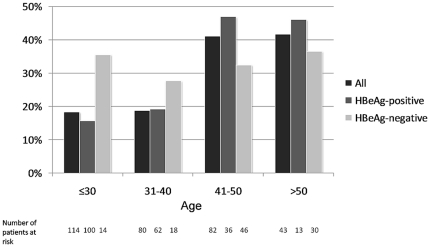
Distribution of significant liver fibrosis (Ishak fibrosis score ≥3) among different age groups.

The distribution of liver necroinflammation and fibrosis is shown in Figures 1 and 2. Significant necroinflammation and fibrosis were found in 54.2% (57.8% for HBeAg-positive, 47.2% for HBeAg-negative) and 27.6% (24.6% for HBeAg-positive, 33.3% for HBeAg-negative) of patients respectively. Altogether 88 patients had significant fibrosis, of which 32 (36.4%) had an ALT level of 1 to 2×ULN. Six patients (5 HBeAg-positive, 1 HBeAg-negative) had histologic evidence of cirrhosis. The distribution of significant liver fibrosis among different age groups is shown in Figure 3. For the whole study population, the proportion of significant fibrosis increased from 18.3% in patients with an age of 30 years or less to 41.9% in patients older than 50 years. HBeAg-positive patients show an increasing prevalence of significant fibrosis with age (p = 0.001). A similar trend was seen in HBeAg-negative patients, except for the group of age less than 30 years (n = 14).

**Figure 4:**
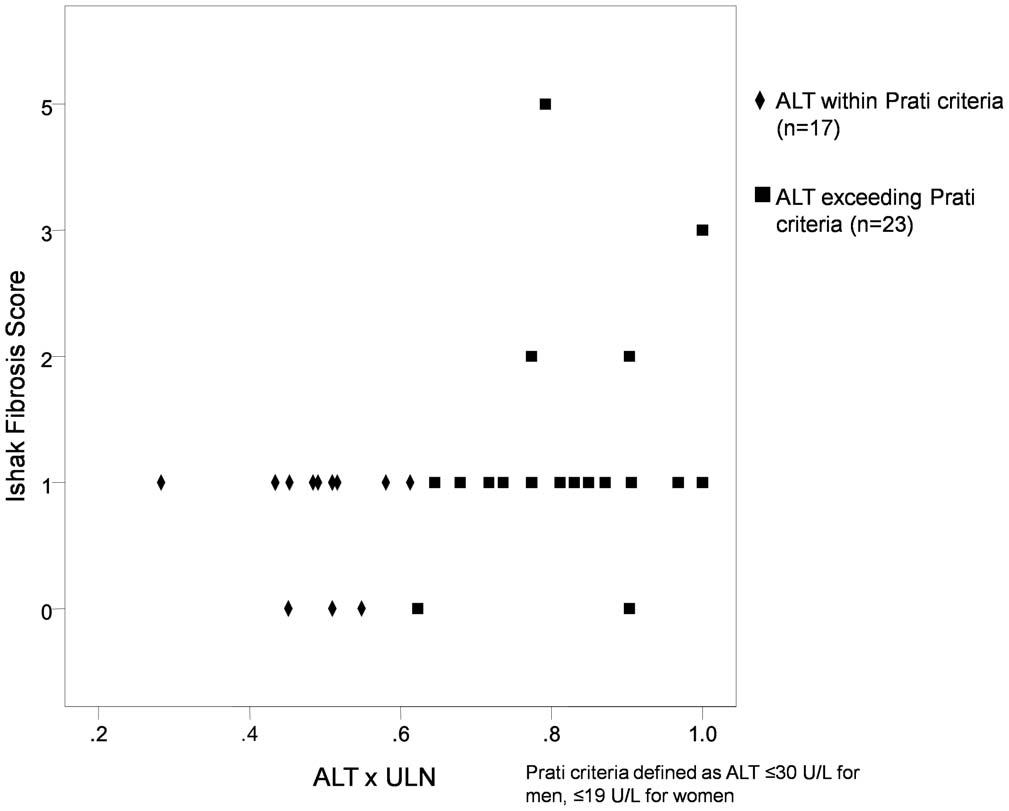
Comparison of Ishak fibrosis scores among HBeAg-positive patients with normal ALT (p = 0.024).

Patients were further analyzed according to different stages of disease. Among the 40 HBeAg-positive patients with a normal serum ALT, 9 (22.5%) had significant necroinflammation and of these 9 patients, 2 (22.2%) had significant fibrosis. These 9 patients had a median age of 29 (range: 17 to 42) and a median ALT of 26 (range: 18 to 53). However, only 17 patients (42.5%) had an ALT level within the normal reference ranges suggested by Prati et al [17] (i.e. 30 U/L for men and 19 U/L for women). Among these 17 patients, only 2 (11.8%) showed significant necroinflammation and none had significant fibrosis. A comparison of fibrosis scores between those with ALT levels within the Prati criteria and those exceeding the Prati criteria among all 40 patients is shown in Figure 4. Patients with ALT levels exceeding the Prati criteria were more likely to have significant fibrosis (p = 0.024).

**Figure 5:**
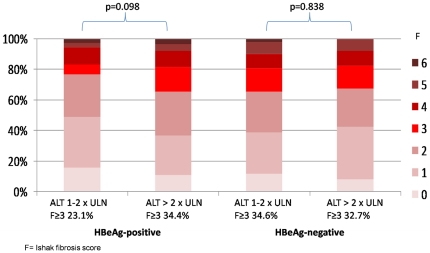
Comparison of Ishak fibrosis scores between ALT 1–2×ULN and ALT >2×ULN in both HBeAg-positive and HBeAg-negative patients.

One hundred and seventy-one HBeAg-positive patients had an elevated ALT. Univariate analysis of showed that age (p = 0.015), albumin (p = <0.001), ALT (p = 0.01), AST (p = 0.005) and platelet count (p = 0.012) were associated with significant necroinflammation, while age (p<0.001), ALT (p = 0.013), AST (p<0.001) and platelet count (p = 0.005) were also associated with significant fibrosis. The multivariate analysis of clinical parameters independently associated with significant necroinflammation or fibrosis is shown in Table 3. A lower serum albumin (p = 0.001) and platelet count (p = 0.037) were independently associated with significant necroinflammation, while older age, higher AST and lower platelet count were independently associated with significant fibrosis (p = 0.007, 0.047 and 0.045 respectively). A higher ALT was not independently associated with significant fibrosis (p = 0.345). The Ishak fibrosis scores among the 78 patients with an ALT level of 1–2×ULN were compared with the 93 patients with an ALT of more than 2×ULN (Figure 5). There was no significant difference noted (p = 0.098). After revising the ULN of ALT according to the Prati criteria, the fibrosis scores of patients with ALT 1–2×ULN (n = 33) were compared with patients having ALT more than 2×ULN (n = 161). Again, there was no significant difference (p = 0.106).

**Table 3:** Clinical parameters predictive of significant liver necroinflammation and fibrosis by multivariate analysis.

		Clinical Parameter	p value	Odds ratio	95% Confidence Interval
HBeAg-positive with elevated ALT (n = 171)	Knodell HAI ≥7 (n = 113)	Albumin (g/L)	0.001	0.828	0.738–0.929
		Platelet (×10^9^/L)	0.037	0.993	0.986–1.000
	Ishak Fibrosis score ≥3 (n = 50)	Age (years)	0.007	1.048	1.013–1.084
		AST (U/L)	0.047	1.108	1.000–1.036
		Platelet (×10^9^/L)	0.045	0.992	0.985–1.000
HBeAg-negative (n = 108)	Knodell HAI ≥7 (n = 51)	Albumin (g/L)	0.003	0.792	0.680–0.923
		DNA (log IU/mL)	0.004	1.741	1.194–2.540
	Ishak Fibrosis score ≥3 (n = 36)	Platelet (×10^9^/L)	0.015	0.988	0.978–0.998
		DNA (log IU/mL)	0.020	1.513	1.066–2.147

From the univariate analysis of 108 HBeAg-negative patients, albumin (p<0.001), ALT (p = 0.038), AST (p = 0.013) and HBV DNA (p = 0.001) were associated with significant necroinflammation, while only platelet count (p = 0.005) and HBV DNA (p = 0.002) were associated with significant fibrosis. The multivariate analysis of clinical parameters independently associated with significant necroinflammation and fibrosis in HBeAg-negative disease are shown in Table 3. A lower serum albumin (p = 0.003) and a higher serum HBV DNA level (p = 0.004) were independently associated with significant necroinflammation, while a low platelet count (p = 0.015) and an increased HBV DNA level (p = 0.020) were independently associated with significant fibrosis. Increasing age and elevated ALT levels were not independently associated with significant fibrosis (p = 0.779 and 0.544 respectively).The Ishak fibrosis scores among patients with an ALT level of 1–2×ULN (n = 52) were compared with those having an ALT level of more than 2×ULN (n = 52) (Figure 5). There was again no significant difference (p = 0.838). After the Prati criteria revision, based on the 10 and 98 patients with ALT 1–2×ULN and more than 2×ULN respectively, fibrosis score also had no significant difference (p = 0.485).

## Discussion

Various treatment guidelines require a certain ALT threshold for treatment decisions. Both the guidelines from the American Association for the Study of Liver Diseases (AASLD) [12] and the Asia-Pacific Association for the Study of the Liver (APASL) [13] only consider treatment when ALT is more than 2×ULN, while treatment is optional for ALT 1 to 2 times×ULN with liver biopsy recommended. Using such an ALT threshold is open to debate, especially when previous studies had shown CHB patients with a normal ALT at the upper range had an increased risk of long-term cirrhotic complications and HCC [25], [26]. Our present study investigated the association of various routinely available clinical parameters, including ALT, with liver histology for all three phases of disease in CHB: the immune tolerant phase, the immune clearance phase and HBeAg-negative disease.

In the present study, we first examined the role of normal ALT levels in predicting liver histology. 22.5% (9 out of 40) of HBeAg-positive patients with a normal ALT had significant histologic abnormalities present. A significant proportion of these patients had abnormal ALT levels using the reference ranges suggested by Prati et al [17]. Therefore, the findings of our study support lowering the currently accepted reference ranges for ALT. In fact, an Asian study based on 1105 healthy individuals also concluded the ULN of ALT should be lowered, this time to 33 U/L for men and 25 U/L for women [27]. In addition, patients with an ALT above 0.5×ULN (defined as 53 U/L for male and 36 U/L for female in that study) were already shown to at an increased risk of cirrhotic complications [25]. Thus, HBeAg-positive patients with a serum ALT at the upper range of the traditionally-accepted normal ranges may in fact be in the immune clearance phase, and hence leading to significant fibrosis on liver histology. A previous study found 39% of HBeAg-positive patients with a normal ALT to have significant fibrosis [16]. Further population studies in different ethnic groups are needed to define the exact suitable reference ranges for ALT.

We then continued to investigate whether there was any difference in liver histology in patients with different levels of elevated ALT. Several studies had demonstrated that there was no good association between ALT levels and fibrosis [16], [21], [28]. Our current study also found that the degree of ALT elevation was not associated with significant changes in necroinflammation or fibrosis for both HBeAg-positive and -negative patients. In addition, there was no significance difference in fibrosis staging for both groups of patients with an ALT level of 1–2×ULN and more than 2×ULN (p = 0.098 and p = 0.838 respectively). Therefore, using the ALT threshold of more than 2×ULN in selecting patients for treatment has the potential of not treating patients with significant fibrosis or cirrhosis. However, this finding should be further confirmed by studies with larger number of HBeAg-positive and -negative patients with different ALT levels.

Increasing age was independently associated with significant fibrosis in HBeAg-positive patients with an elevated ALT, which is consistent with previous studies [16], [19], [29]. In our present study, age had no association with fibrosis in HBeAg-negative disease, with a substantial proportion of young patients of age 30 or less having significant fibrosis (35.7%, 5 out of 14). While this could be related to the small sample size for these patients, another possible explanation is that the majority of HBeAg-negative patients in our study (96.3%, 105 out of 108) had an elevated ALT (median level 90 U/L) and HBV DNA (median level 5.71 log IU/mL). This may represent a special group of patients who underwent HBeAg seroconversion at a relatively younger age (median age of HBeAg seroconversion in our locality is 35 years [25]) and yet having a more serious disease as reflected by high ALT and HBV DNA levels. Regardless of this, a study in Asia had shown the prevalence of significant fibrosis among HBeAg-negative patients with the age of less than 25 years to be 17% [29], which is much higher than figures reported in Europe [20]. This can be explained by Asian patients acquiring the infection perinatally, with liver injury starting early in life.

For HBeAg-negative disease, increased HBV DNA levels were independently associated with significant fibrosis. The findings are in line with the suggestion of suppressing HBV DNA permanently as a treatment end point [30], especially when long-term viral suppression has been shown to reverse histologic damage [31], [32]. Serum HBV DNA levels had no association with fibrosis in the immune clearance phase, which can be explained by an extensive immune-mediated response leading to low viremic levels despite significant abnormalities on histology [33]. Although a study from Taiwan showed that normal ALT levels in HBeAg-negative disease are associated with a good prognosis and recommended treatment initiation only for patients with ALT levels more than 2×ULN [14], HBV DNA measurements were not incorporated in that study. A large population study has shown elevated HBV DNA levels in noncirrhotic HBeAg-negative patients with normal ALT to be associated with an increased risk of HCC [7].

Among HBeAg-positive patients with elevated ALT (n = 171), both elevated AST and low platelet count were independently associated with significant fibrosis, although the p values were only borderline significant (p = 0.047 and 0.045 respectively). These findings are however consistent with a previous study showing that both AST and platelet count are associated with significant fibrosis, from which the AST to platelet ratio index (APRI) was derived [34]._In addition, our current findings are also in line with our previous study in which AST and platelet count were used to create a model to predict significant fibrosis [22]. Another model known as the APGA (AST/platelet/GGT/AFP) score showed promising results when correlating with liver stiffness measured by transient elastography in CHB. Further studies correlating with actual histologic specimens would be needed to determine its clinical applicability [35].

There are several limitations in our study, including its retrospective nature, the different biopsy methods used and the lack of HBeAg-negative patients with normal ALT. Although the differences in inclusion criteria would result in selection bias, our present study was able to recruit patients from different phases of disease, thus making the study more representative of the whole CHB patient population. HBV genotype and the presence of the core-promoter mutation were not checked in our present study, which could be important since previous small-scale studies found genotype C and the core-promoter mutation to be associated with significant histologic abnormalities [19], [36]. However, the determination of HBV genotype and core promoter mutations requires more advanced molecular diagnostics, and may not be available for routine clinical practice. Since serum ALT and HBV DNA levels tend to fluctuate with time [37], the measurement of each clinical parameter at several time points might be more representative. An average ALT obtained by integral calculus would be one such method [38]. However, whether an average value or a single value at the time of biopsy would have better histologic correlation and prognostic determination in a cross-sectional study would require further evaluation. Our study also lacked clinical information concerning the patient's metabolic profile i.e. the presence of diabetes mellitus or insulin resistance. Nevertheless, recent studies focusing on Chinese CHB patients did not show any association between metabolic factors and liver disease severity [39], [40].

In conclusion, ALT is not a useful marker for the decision of commencing antiviral therapy in CHB because of its poor correlation with significant liver injury in both HBeAg-positive and -negative patients. Patients with ALT levels less than 2×ULN should be considered for possible treatment if histology, or other non-invasive assessment such as transient elastography, shows significant fibrosis. HBeAg-positive CHB with a normal ALT might already have significant histologic abnormalities, which supports the lowering of the current ALT reference ranges. Increased HBV DNA levels and low platelet count are associated with significant fibrosis in HBeAg-negative disease.
